# Understanding the financial barriers to treatment among individuals with opioid use disorder: a focus group study

**DOI:** 10.1186/s12954-024-01133-4

**Published:** 2024-12-20

**Authors:** Precious Anyanwu, Olajumoke A. Olateju, Vaishnavi Tata, Tyler Varisco, Lauren R. Gilbert, Motolani E. Ogunsanya, E. James Essien, Michael L. Johnson, J. Douglas Thornton

**Affiliations:** 1 Department of Pharmaceutical Health Outcomes and Policy, University of Houston College of Pharmacy, Health 2 Building, 4349 Martin Luther King Boulevard, Houston, TX 77204-5047 USA; 2 Prescription Drug Misuse and Education Research (PREMIER) Center, University of Houston College of Pharmacy, Health 2 Building, 4349 Martin Luther King Boulevard, Houston, TX 77204-5047 USA; 3 Department of Health Systems and Population Health Sciences, University of Houston College of Medicine, Houston, TX 77204 USA; 4 Department of Pharmacy, Clinical and Administrative Sciences, University of Oklahoma Health Science Center, Oklahoma City, OK 73117 USA

**Keywords:** Opioid use disorder, Financial barriers, Focus group, Financial distress, MOUD

## Abstract

**Introduction:**

Despite the established effectiveness and relatively widespread availability of Medications for Opioid Use Disorder, individuals seeking treatment frequently encounter various structural and social barriers, including costs of treatment. This study aimed to understand the financial barriers that affect treatment continuation in individuals with opioid use disorder (OUD).

**Methods:**

In this qualitative study, seven semi-structured in-depth focus group interviews were conducted among 28 participants in treatment for OUD. Basic demographic information were collected in a pre-focus group survey. Focus group interviews were conducted from December 2021 to February 2022. A moderator guide was used to facilitate the discussion. Transcripts were managed using ATLAS.ti© v7. Data collected from the focus groups underwent deductive thematic analysis.

**Results:**

Data saturation was reached in 7 focus groups with a total of 28 participants (17 [60.7%] women; 27 [96.4%] white; 24 [85.7%] non-Hispanic). All three medications for OUD were represented (18 [64.3%] buprenorphine and naloxone, 5 [17.9%] methadone, 3 [10.7%] naltrexone, and 2 [7.1%] buprenorphine) and the time in treatment ranged from 1 month to over 36 months. Nearly all participants (27 [96.4%]) indicated a financial barrier that led to delayed treatment initiation and treatment retention. Two themes were identified from the focus group interviews: (1) OUD treatment-related financial burden- the direct and indirect cost associated with the treatment, and (2) psychosocial effects associated with the cost of OUD treatment -the mental, emotional, and social effects of the disease.

**Conclusions:**

Most participants described the desire and need for resources to offset the unaffordable cost that inhibits treatment initiation and retention. Further work is required to help identify individuals susceptible to financial barriers that can lead to early discontinuation in treatment.

**Supplementary Information:**

The online version contains supplementary material available at 10.1186/s12954-024-01133-4.

## Introduction

In 2020, nearly 91,800 persons in the United States died from a drug-involved overdose, and about 75% of those deaths involved an opioid [15]. In the face of the third wave of the ongoing opioid crisis, federal agencies such as the United States Department of Health and Human Services (HHS) have prioritized access to better addiction treatment and recovery services [1, 35]. Part of this strategy involves increasing access to medications for opioid use disorder (MOUD) [27]. Methadone use is associated with reduced rates of drug use and death among patients with opioid use disorder (OUD) [23]. Buprenorphine and naltrexone have also shown similar progress in treating patients with OUD and have the added convenience of being prescribed in office-based settings [4, 5, 21]. Although prevailing treatment guidelines recommend MOUD, a study based on the 2019 National Survey on Drug Use and Health (NSDUH) reported that only 27.8% of individuals needing OUD treatment received treatment with a MOUD [24].

Many factors exist that inhibit access to MOUDs, such as stigma, cultural barriers, lack of readiness to quit, negative perceptions surrounding treatment effectiveness or side effects, fear of legal implications, limited access due to low facility and clinician uptake, and logistic, geographical and financial barriers amongst others [6, 12, 14, 17, 18, 26, 29, 36]. The 2020 NSDUH Annual National Report reported that 20% of people aged 12 and older with a substance use disorder (SUD) in the past year who did not receive treatment but perceived the need for treatment cited a lack of health insurance or ability to afford treatment as the primary cause [3]. This problem is further compounded by the finding that 61% of opioid treatment programs (OTPs) are operated by for-profit organizations that reserve treatment for those who either have health insurance the program accepts or can pay out-of-pocket [2]. These factors exacerbate existing barriers to seeking treatment for OUD, but the financial burden that persists for a patient entering or retaining treatment is understudied.

Using a deductive thematic analysis, this study aims to understand the financial burden individuals face with OUD and its effects on their recovery and daily life. While financial costs are often cited as barriers to accessing MOUD, much of the literature focuses on direct treatment expenses, leaving the broader financial burden, such as indirect costs from lost wages and transportation less documented. These indirect costs, which can significantly impact treatment adherence and long-term success, are frequently underreported. Existing studies suggest that financial incentives or making treatment free can improve MOUD uptake, highlighting the need to further examine how both direct and indirect financial burdens affect treatment accessibility and outcomes. Findings from this study may support the strengthening of research and policies that measure the degree to which treatment successes are due to financial toxicity.

## Methods

### Study design and research team

**Fig. 1:**
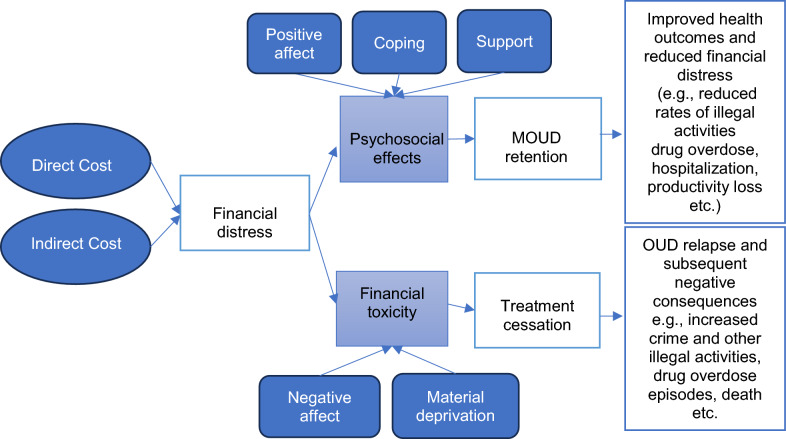
Conceptual framework of opioid use disorder treatment-related financial toxicity. Study themes are in light blue boxes while the subthemes are in darker blue boxes

This qualitative study used a deductive thematic analysis of focus group discussions to identify how individuals in treatment for OUD are affected by the costs associated with treatment. This analytical approach was utilized because this study was driven by theories from literature and concepts from key stakeholders including clinicians, OUD community program partners, patients with lived and living experience, and research experts (Fig. 1) [8]. For this study, costs include the direct (i.e., patient out-of-pocket costs for outpatient medical services, hospitalizations, and outpatient pharmacy costs), indirect (e.g., transportation to receive treatment, housing instability, productivity loss, opportunity costs, and time spent coordinating or waiting for care), and the psychological and social aspects associated with the cost of treatment (e.g., impacts on mental, emotional, and spiritual well-being due to OUD, behavioral, legal, and psychological effects of the disease, and support costs from friends and family) (Table 2). The research team consisted of members from the Prescription Drug Misuse Education and Research (PREMIER) Center housed within the University of Houston College of Pharmacy and a faculty member from the University of Houston College of Medicine. The Premier center comprises faculty members, fellows, graduate students, and clinicians with a background in health service research and prevention with expertise in qualitative and quantitative research projects regarding drug misuse and prevention. The research team had no established relationship with the study participants prior to the study commencement. The University of Houston Institutional Review Board approved the study protocol. The 32-Item Consolidated Criteria for Reporting Qualitative Research (COREQ) reporting guideline was followed [33].

### Setting and participants

The sample in this study was identified via purposeful sampling. The research team recruited participants in treatment for OUD who were a part of a community program for people with OUD or part of a local recovery housing community. These programs primarily attract individuals committed to addressing their OUD and provide structured support services that facilitate recovery. The community program aims to help patients navigate the complicated recovery journey by combining medical and behavioral support services for optimal engagement and retention in outpatient treatment. The recovery housing community aims to bridge the gap between inpatient treatment and independent living.

The research team created and disseminated a recruitment flyer containing information about the study, reasons for conducting the study, and what participants would expect if they elected to be a part of the study. Interested individuals contacted the research team and were notified via email or text if they were eligible for inclusion. Participants were eligible if they were at least 18 years of age, currently receiving a MOUD, and spoke English fluently. Recruitment happened on a rolling basis between December 2021 and February 2022. All necessary information about the study and interviewer(s) were provided to participants. Participants gave electronic consent for the virtual focus groups and written consent for in-person focus groups. The recommend number of participants in virtual focus groups is 3–6 participants and 4–8 for in-person focus groups [9, 19, 30, 34]. These recommendations were implemented when participants were recruited for the focus groups. Selected participants received a $50 gift card as an honorarium for participation in the focus groups.

### Data collection

All focus groups were guided by a predeveloped moderator guide that contained questions designed to explore how the cost of treatment impacted participants’ recovery and daily life throughout their treatment for OUD. The questions were derived from discussions with key opinion leaders and literature. Focus groups, which lasted for approximately 60 min, were held on a rolling basis between December 2021 and February 2022. Virtual focus groups were held via Zoom® conferencing software and were audio and video recorded. In-person focus groups were only audio recorded. The interview questions were modified based on participant’s feedback. Focus groups (n = 6) were held until data saturation was reached; saturation is the point in the research process at which no new information was uncovered through data analysis [28]. All focus groups, virtual and in-person, were conducted by the same research team member, a female researcher who has training in qualitative data collection. Upon completion of each focus group, all audio recordings were transcribed with Rev® software to result in transcripts for analysis.

### Data analysis

The resulting transcripts were uploaded into ATLAS.ti© v7, a qualitative data management software for analysis [13]. Two members of the research team then followed the steps for analysis below. Two research team members followed the analysis steps outlined below, with any discrepancies resolved by a senior researcher to ensure accuracy and consistency.

1. *Data familiarization* Each member independently read the focus group transcripts and noted initial observations.
2. *Generating codes* The members discussed the transcripts' significant aspects and pertinent recurring themes. Then, each member reexamined the data from the transcripts and generated their own codes. A faculty member served as the arbitrator in a discussion between the research team members to reconcile the two distinct lists of codes. This procedure produced the final codebook.
3. *Searching for sub-themes* After generating the codebook, the two members of the research team gathered to organize the codes into overarching themes.
4. *Reviewing sub-themes* The themes were discussed, compiled, and reconciled in order to generate a thematic map. In addition, this study’s software, ATLAS.ti V722, can generate semantic networks.
5. *Deciding on sub-hemes* After determining the themes, the two members of the research team responsible for coding and the arbitrating faculty member reviewed and appropriately named the themes.
6. *Producing the report* The final report included seven transcripts that had been coded, merged, and reconciled, along with the codebook and themes.

## Results

### Characteristics of study participants

Data saturation was achieved in seven focus groups with 28 participants. A total of 29 participants completed the focus group consent form, with one not participating due to scheduling changes. Data from a pre-focus group survey that most of the participants were female (61%), white (96.4%), non-Hispanic (89%), and between 30 and 49 years of age (71%). The participants’ other characteristics, including their level of education, employment status, type of MOUD, and duration of time in treatment, are detailed in Table 1. A total of 27 (96.4%) indicated that a financial barrier led to delayed treatment initiation or inhibited treatment retention.

**Table 1:** Demographics of participants from seven focus groups

Characteristic	No. (%) (N = 28)
*Sex*	
Male	11 (39.3)
Female	17 (60.7)
*Race*	
White	27 (96.4)
Other	1 (3.6)
*Ethnicity*	
Non- Hispanic	24 (85.7)
Hispanic	4 (14.3)
*Age, years*	
18–29	4 (14.3)
30–39	12 (42.9)
40+	12 (42.9)
*Level of Education*	
Some High School	1 (3.6)
High School graduate or GED equivalent	5 (17.9)
Some College	20 (71.4)
College Graduate	2 (7.1)
*Employment Status*	
Unemployed	15 (53.7)
Part-time or temporary worker	4 (14.3)
Full-time Employee	9 (32.1)
*Type of MOUD*	
Methadone	5 (17.9)
Naltrexone	3 (10.7)
Buprenorphine	2 (7.1)
Buprenorphine with Naloxone	18 (64.3)
*Time on MOUD*	
0–3 months	8 (28.6)
4–9 months	6 (21.4)
10–12 months	3 (10.7)
≥ 1 year to < 2 years	5 (17.9)
≥ 2 years to < 3 years	2 (17.9)

MOUD, Medication for opioid use disorder

GED, General Educational Development

### Qualitative results

Qualitative analysis of the seven focus groups identified two primary themes (five sub-themes) characterizing the financial barriers to treatment for OUD: *(1) OUD treatment-related financial burden* and *(2) Psychosocial effects associated with the cost of OUD treatment*. Table 2 summarizes these themes, subthemes, codes, and definitions. Table 3 consists of participants' quotes that illustrate the subthemes and code. These tables provide an overview of the results from the focus groups, while the following sections concentrate on the critical finding from each theme.

**Table 2:** Taxonomy of concepts describing financial barriers to treatment for opioid use disorder

Theme	Subtheme	Codes	Definition
OUD Treatment-Related Financial Burden The direct and Indirect costs associated with the treatment of OUD	Direct The cost incurred because of treatment for OUD	Treatment Initiation	The ability or inability to initiate treatment due to cost
		Treatment Retention	The ability or inability to remain in treatment due to cost
		Financial Support from Outside Sources	The Ability to remain in treatment is due to financial support from other sources
	Indirect Cost Expenses faced when seeking treatment that is not directly tied to the medical bill	Transportation	Difficulty affording or maintaining transportation to receive treatment
		Housing	Difficulty maintaining housing and the ability to afford treatment
		Lost Wages	Absence from work or lost hours to obtain treatment
		Administrative Burden	Time spent coordinating care or waiting for care
Psychosocial Effects Associated With The Cost of OUD Treatment The mental, emotional, social, and spiritual effects of disease	Coping Conscious and unconscious strategies used to overcome problems and difficulties associated with the cost of OUD treatment	Early Discontinuation in Treatment	Discontinuing treatment due to the cost
		Criminality	Behavior that is contrary to or forbidden by criminal law
		Drug Diversion	Illegal distribution of prescription drugs and their use for purposes not intended by the prescriber
		Non-Pharmacological Treatment	Non- Pharmacological methods used to maintain sobriety
	Affect The emotional reaction due to the cost of treatment	Frustration, Anger, or Outrage	The feeling of frustration, anger, or fear because of the inability to afford treatment
		Worry, Anxiety, or Fear	The feeling of panic and stress because of the inability to afford treatment
	Support The emotional or social support or lack of emotional support that contributes to treatment retention or discontinuation	Family Support	Family support or lack of support
		Workplace Support	Workplace support or lack of support for treatment
		Support from Others	Support or lack of support from others

**Table 3:** Key quotes demonstrating themes

Theme	Subtheme	Quote
OUD Treatment-Related Financial Burden	Direct	[A friend of the participant] “He was like, ‘I need to get my money right,’ is what he said to me. ‘Let me get a few things in order, and I'm going to do that so that I can afford to pay for my medication.’ He’s dead.”
		“Recently, in these past couple of months, I lost my regular job. And when that happened, I kind of got screwed on the medication aspect of that, so.”
		“I had insurance, but the insurance usually doesn’t cover it, my insurance at least. But yeah, I'm grateful they got the state to fund it.”
	Indirect Cost	“My truck broke down, and I had to spend $400 in order to make it to my appointment. And so I had to Uber my way up there for the first two months of that happening until I got enough money to fix my truck. So if you do the math, that’s almost… That’s close to a grand.”
		“I need a roommate because I can’t pay rent. I can’t pay rent because my medication is $480. That’s over half of my rent
		“You'd be waiting for like three hours to get through it. And they didn’t care if you had a job or not. I had to go to work. They wouldn’t put you ahead of people that didn’t have jobs or anything like that. And I can’t tell… like I literally got fired from one of my jobs because I was late so much because I was at the clinic.”
		“The places that you can go, without money, I mean they have six-month waiting lists. By the time that comes around, I'll probably be OD'd. The last six months of using, I had OD'd and ended up in the hospital four times, so the likelihood of me making it another six months is very unlikely.”
Psychosocial Effects Associated with the Cost of OUD Treatment	Coping	“I didn’t have a choice. I had to stop taking Suboxone.”
		“Anything I could get my hands on, I'm going to sell it, either to get my fix, or to pay for the weeks’ worth, or to get my meds. Whatever I'm a part of at that point in time, that’s what I'm what I'm going to do.”
		“Breaking the law, and like I said, one of the best ways to do it, if you can, is to maybe lie to your doctor, get some medications, get a number of medications, take the ones that you need, and have some to pay off for whatever you got on the streets.”
		“If I'm not working, then I don’t get it. That’s the problem right now. If I don’t have the money, I don’t have anybody to help me. So I just don’t get the prescription, and I go without it, and I have to try to make myself really focus and go to meetings and do stuff like that during that time that I'm out of it.”
	Affect	“I have a lot of anxiety over it [cost of treatment]. Like I said, it’s on a week-to-week basis. I'm trying to figure out how I'm going to pay for it. It’s like you're trying to do the right thing, and it’s so much harder than doing the wrong thing. It'd be so much easier to go back and do what you've been doing, but when you try to change, it’s a whole bunch of extra stuff comes with it, and it can be frustrating.”
		“I worry about how you see like prescription drug costs and things go up. I wonder… I worry about in my lifetime that it will escalate to the point that I won’t be able to afford it. And I'm really afraid of that because, like with methadone, it’s till you're dead basically you'll be on it. And I'm like, so how am I going to afford this. Because as I get older, it’s just going to get more and more expensive. So it does make me nervous.”
	Support	"No, I can’t go to treatment. I literally am holding up my entire family. If I leave my job, my everything falls apart."
		“If I can’t go to work, am I going to go to that meeting? Or am I going to get kicked out of this program if I don’t go to this meeting?”
		“I'm grateful for the programs, but I think there’s a lot of help out there, but people don’t know about these resources. It needs better public attention, I guess you could say that.”

OUD, opioid use disorder

#### OUD treatment-related financial burden: direct cost

When addressing cost barriers to treatment in outpatient settings, participants recalled that the direct cost of treatment (the cost associated with their medical bill) was considered unaffordable and inhibited initiating or retaining treatment in outpatient settings. One participant mentioned the direct cost as the sole reason for not starting treatment. “Well, to be honest, this time, I didn’t go to treatment because I didn’t think I could afford it. Financially, it wasn’t an option for me. I didn’t go simply because of that.” Regarding the inability to remain in treatment due to cost, one participant stated:

“When I first found out how much it was going to be [medication] when I first started paying out of pocket, I had to walk away. Because I didn’t have that money in my pocket to be able to pay, so I was kind of sour. Like, ‘Damn, here I am trying to get clean, and you're charging an arm and a leg. [S***], my dope is cheaper than what I had to pay for Suboxone.”

Another participant shared this perspective when comparing the cost of medication treatment to the cost of illicit opioid use: “It’s cheaper to stay high than to get on your medication and do the right thing.” However, many participants asserted that their ability to remain in treatment was due to financial support from public or private insurance or grants. One participant stated, “If it wasn’t for the state funding, how can I afford to go to treatment and go pay all this money when I just spent all my money on drugs and alcohol?”. With the reliance on external financial support being a significant factor in retaining treatment, loss of that support was reported to contribute to reverting to illicit opioid use. One participant stated, “Because I lost my insurance, and it wasn’t affordable. I went straight back to shooting heroin because I couldn’t deal with the withdrawals. I’ve been in a situation where I've lost my treatment due to financial issues.”

#### OUD treatment-related financial burden: indirect cost

Participants also indicated that the indirect costs of treatment (expenses faced when seeking treatment unrelated to the medical bill) contributed to the financial burden. Participants often mentioned issues with transportation to treatment appointments, housing, lost wages, and the administrative burden. One participant receiving methadone stated:

“I was forced to find rides to a clinic 40 minutes away every day, whether it was family or friends, paying someone $40 to take me down there, or paying Uber $50. That was every day except Saturday. There was a huge, huge burden with that financially, [and] time-wise. And then there were times where I just simply couldn’t get down there and had to just miss my dose because I couldn’t find someone or I didn’t have the money to pay someone to take me down there.”

In contrast, participants also stated that some treatment providers are aware of transportation issues and have provided additional support and services to attend appointments virtually to help minimize the financial burden. One participant stated, “Good thing is that my doctor actually is doing virtual appointments now.” Housing costs often posed a significant barrier to maintaining treatment rather than initiating it, as some participants described having to prioritize between covering housing expenses and affording treatment, which affected their ability to stay engaged in care. Focus groups often noted having to choose between housing expenses and treatment, which influenced their ability to continue MOUD. A participant stated, “I've lost places to live over trying to get money in my addiction to get Suboxone, as opposed to getting dope. I've lost places to live because I wasn’t able to pay them because I was paying for my Suboxone.”

#### Psychosocial effects associated with the cost of OUD treatment: affect

In psychology, affect is the underlying sensation of feeling, emotion, or mood [16]. Emotional responses were prevalent throughout the focus groups when participants discussed the cost of treatment of OUD. Some of the negative emotional responses expressed during the focus groups were feeling of frustration, anger, anxiety, and fear, as demonstrated by one participant who stated:

“It is a pain in the ass, but I want to stay sober, so I just have to suck it up and do whatever I'm going to do. But I think it’s ridiculous… the price of Suboxone, especially... I mean, we need it. Us addicts, we need that medication, just as somebody that’s diabetic needs their medication. I don’t know. I wish it was a lot cheaper, especially if it’s helping somebody, but the case is it’s not right now. So, I just got to roll with the punches.”

Participants also expressed a worry about not being able to remain in recovery due to the cost of treatment; A participant stated, “I think the only scary thing about it is I have Medicaid right now, but when it runs out, it’s expensive, and when you don’t have it, you withdraw, so that’s going put me into the relapse category.”

#### Psychosocial effects associated with the cost of OUD treatment: coping

Coping behaviors are characterized as deliberate and conscious responses to the demands and emotions of stressful situations [11, 22]. Participants described different strategies used in efforts to afford treatment. Often participants indicated early discontinuation in their treatment to cope with the unaffordable cost, “So, there would be times when I couldn’t pay for the week all right up front, so I would just not go get my medication, and I'd end up using like two days into it.” Many participants indicated that they focus on therapy and utilizing the resources provided by programs. One participant stated, “I would not be sober if it wasn’t for ‘NA’ [Narcotics Anonymous]. I wouldn’t know how to live. It shows you a design for living.”. However, many participants have engaged in risky, dangerous, and illegal measures to afford treatment. Some participants reported engaging in the same criminal activity they previously used to support their illicit opioid use to pay for their treatment. A participant stated.

“So then I was doing crazy stuff to keep my high, stealing cars, stealing 18 wheelers, stealing from Walmart… I never got caught by the cops. I became very good at it, so once I got in recovery, there was a point where I had to get Suboxone…I still had those habits so that I could get my Suboxone. I mean, I'm struggling. What am I going to do? I'm going to do what I know what to do. I know I ain’t got to have a job to make money.”

Drug diversion was also commonly stated as a method used to pay for treatment “Back in my twenties, I would have to sell some of my medication on the streets in order to be able to at least maybe get some of the money to be able to pay for my medication.”

#### Psychosocial effects associated with the cost of OUD treatment: support

Participants indicated social and emotional support or the lack of emotional and social support they received from family or place of employment affected their ability to remain in treatment. When discussing employers, participants indicated it was difficult to stay employed and attend meetings to maintain the aid they receive from grants to pay for treatment. A participant stated, “Employers aren’t really flexible on that. They don’t want an employee who’s going to have to leave, every job they send him on out of town. He is going to have to go 45 min out of his way to go to a clinic to get his medication.” It was also reported that lack of support from family and friends negatively affects their ability to remain in treatment. However, family and friends' assistance allowed participants to focus on staying in treatment. A participant stated, “[without my family] Yeah, I wouldn’t have this house. Wouldn’t have the car. Wouldn’t be able to take care of my dog, who’s my firstborn. Wouldn’t be able to eat.” Support from grant-funded programs also contributed to participants feeling increased autonomy and aided in the ability to maintain treatment.

“I'm fortunate enough that the position I'm in now financially, because I have been given the time to focus on the other aspects of my life, I wouldn’t have to go commit a crime, steal, rob, sell drugs to be able to afford it. Right now, I'm very fortunate that I've had those programs to allow me to build up where I'm at.”

## Discussion

This study is the first, to the research team’s knowledge, to qualitatively examine the effects the cost of OUD treatment has on individual daily life and recovery. Understanding these barriers allows OUD’s treatment resources to be allocated in a manner that grants more expansive access to individuals seeking treatment. The findings from this qualitative research study highlight a significant problem faced by those seeking MOUD.

The theme “OUD treatment-related financial burden” encompassed the subthemes of treatment’s direct and indirect costs. It should be of note that this qualitative research study took place in Texas, one of the 12 states that have not expanded Medicaid coverage under the Patient Protection Affordable Care Act. Medicaid is a federal health insurance program that provides treatment coverage to disabled and low-income individuals. A previous study found that expanding Medicaid substantially increased individuals with OUD receiving psychosocial treatment and MOUD [25]. Expanding Medicaid in Texas would make the out-of-pocket cost of OUD treatment more affordable for many and subsequently allow individuals to allocate funds towards the indirect cost of treatment for OUD. Although many participants found funding for their treatment through grants, these grants are a finite resource, and waiting to access them can be detrimental to an individual with OUD.

A key finding was that to manage the financial burden, many participants reported resorting to criminal activities such as drug diversion and theft to afford treatment. This is not surprising, given the extensive literature linking the financial strain of substance use treatment to criminal behavior [31]. However, these actions highlight a broader issue: the criminal justice system, which absorbs a significant portion of the societal costs of substance use disorder. Publicly funded OUD treatment programs have been shown to reduce these costs by preventing crime and decreasing criminal justice expenditures [20]. In fact, research suggests that increases in treatment utilization led to reductions in criminality, not just through decreased illicit drug use, but also by providing alternatives to crime as a means of financing treatment [10]. This indicates that reducing MOUD costs could have far-reaching societal benefits, not only by improving health outcomes but also by decreasing crime-related costs. Therefore, the association between affordability and crime reduction should be a central consideration in efforts to enhance access to MOUD.

To cope with the financial burden of treatment, several participants reported engaging in criminality, specifically drug diversion and theft, to pay for treatment for OUD. The criminal justice system represents the largest indirect cost share of the societal cost of substance use disorder. However, treatment programs for OUD funded by the public were found to reduce the cost of crime [10, 20]. Although an increase in treatment utilization has been shown to reduce criminality, the causal relationship between illicit drug use and crime is replicated to pay for the treatment due to the unaffordable cost of treatment.

The findings from this study suggest that the financial burden associated with MOUD may hinder individuals with OUD from remaining in treatment and fully benefiting from its established outcomes, such as reduced risks of overdose, hospitalization, and productivity loss. While health insurance coverage and supportive services like transportation and housing assistance are standard methods to ease treatment burdens, many patients still face access challenges. Barriers such as prior authorization requirements and unaffordable out-of-pocket costs can add significant psychosocial strain to the existing challenges of managing OUD. These access issues may vary based on factors like insurance type, OUD severity, and the specific treatment being received (e.g., methadone, which requires higher administrative oversight, versus buprenorphine, which is more accessible in outpatient settings). To improve retention, it may be necessary to stratify patients by risk, considering barriers to access and retention potential. This approach could leverage patient-centered assessments that address clinical, social, and financial dimensions of treatment barriers. For example, a patient-reported outcome instrument could directly measure the perceived impact of financial strain on daily life and recovery, providing a valuable perspective on patient experience in OUD treatment [7]. This framework would enable a more nuanced understanding of the effects of financial toxicity on treatment success, informing targeted support to enhance patient outcomes [7].

This study has several limitations. The findings may be subject to selection bias, recall bias, and social desirability bias, limiting their generalizability. Purposeful sampling was used to recruit participants from a community program and a local recovery housing community, which typically attract individuals motivated to seek treatment and those with access to structured support services. Consequently, our sample may not fully represent the broader OUD population, especially those facing additional barriers to care or lacking similar support. Non-English speakers were excluded, potentially limiting the findings’ applicability to diverse linguistic groups and the unique sociodemographic and socioeconomic challenges they face. Additionally, the sample may not be representative of individuals receiving OUD treatment in rural areas, regions with less publicly funded treatment, inpatient settings, or countries outside the United States. For example, recent additions of methadone and buprenorphine to Australia’s Pharmaceutical Benefits Scheme (PBS) aim to reduce financial barriers to MOUD, highlighting variations in access and funding across countries [32]. Future research should aim to include a more varied OUD population to improve generalizability and address these limitations.

## Conclusion

In this qualitative study, participants with OUD described their desire and need for resources to offset the cost of treatment. This financial strain not only affected their psychosocial well-being but also led some to resort to criminal activities as a means of addressing the burden of treatment expenses. The inability to remain in treatment increases the risk of treatment failure and adverse clinical outcomes, including death. These findings underscore the importance of research and policy initiatives aimed at examining the extent to which treatment outcomes are influenced by financial toxicity, potentially guiding interventions to improve accessibility and success rates for those in need.

## Supplementary Information

Additional file 1.

## Data Availability

Data is available upon request to the corresponding author.
